# The ASC Module: A GPU Memory-Efficient, Physiology-Aware Approach for Improving Segmentation Accuracy on Poorly Contrast-Enhanced CT Scans—A Preliminary Study

**DOI:** 10.3390/bioengineering12090974

**Published:** 2025-09-12

**Authors:** Zuoyuan Zhao, Toru Higaki, Yanlei Gu, Bisser Raytchev

**Affiliations:** Informatics and Data Science Program, Graduate School of Advance Science and Engineering, Hiroshima University, 1-4-1 Kagamiyama, Higashi-Hiroshima, Hiroshima 739-8527, Japan; m242740@hiroshima-u.ac.jp (Z.Z.); guyanlei@hiroshima-u.ac.jp (Y.G.); bisser@hiroshima-u.ac.jp (B.R.)

**Keywords:** GPU memory-efficient, image segmentation, CT images, poor/non-contrasted CT, spatial structure, deep learning

## Abstract

At present, some aging populations, such as those in Japan, face an underlying risk of inadequate medical resources. Using neural networks to assist doctors in locating the aorta in patients via computed tomography (CT) before surgery is a task with practical value. While UNet and some of its derived models are efficient for the semantic segmentation of optimally contrast-enhanced CT images, their segmentation accuracy on poorly or non-contrasted CT images is too low to provide usable results. To solve this problem, we propose a data-processing module based on the physical–spatial structure and anatomical properties of the aorta, which we call the Automatic Spatial Contrast Module. In an experiment using UNet, Attention UNet, TransUNet, and Swin-UNet as baselines, modified versions of these models using the proposed Automatic Spatial Contrast (ASC) Module showed improvements of up to 24.84% in the Intersection-over-Union (IoU) and 28.13% in the Dice Similarity Coefficient (DSC). Furthermore, the proposed approach entails only a small increase in GPU memory when compared with the baseline models.

## 1. Introduction

Advances in neural network technology have made many tasks that once required human experts partially automatable. Assisting physicians with making diagnoses is one such task. In countries with aging populations such as Japan, the use of deep learning models is considered a promising way to mitigate healthcare labor shortages and constrained budgets. In fact, some deep learning models have already been used to solve various medical problems, including in the analysis of CT images [1,2] and mitigating the limitations of CT imaging under adverse conditions [3].

Convolutional neural networks (CNNs) were the dominant paradigm in the computer vision field throughout the 2010s. Starting with AlexNet [4] in 2012, successive architectures, such as VGG [5], GoogLeNet [6], and ResNet [7], continually updated the state of the art in image classification and recognition. Early attempts at semantic segmentation involved adapting these CNNs to a sliding window paradigm, in which each local patch is classified in turn. Although this approach demonstrated that deep networks can learn pixel-level features, the heavy overlap between neighboring windows caused massive computational redundancy and very low throughput.

To eliminate such inefficiency, a new generation of end-to-end segmentation networks emerged. Modern research typically traces this lineage back to Fully Convolutional Networks (FCNs) [8], followed by the encoder–decoder UNet [9] and its derived models, most notably Attention UNet [10]; TransUNet [11], which integrates Vision Transformer (ViT) [12] layers employing self-attention [13]; and Swin-UNet [14], a pure Transformer UNet developed on the basis of the Swin Transformer [15]. These architectures have been widely adopted for the segmentation of computed tomography (CT) images.

In our experiments, these models were found to perform well when CT scans are optimally contrast-enhanced; however, their performance degrades markedly when the contrast is insufficient or absent. As contrast agents cannot always be administered (e.g., due to patient contraindications or in resource-limited settings), it is necessary to develop models that can reliably localize and segment the aorta under low-contrast conditions.

Although redesigning the architecture’s backbone or adding extra inputs/parameters may alleviate this problem, such changes often increase GPU memory consumption and hinder deployment. A more practical alternative is to enhance existing models with lightweight components, rather than making radical architectural changes.

Fully 3D segmentation networks capture rich volumetric context, but the cubic growth of the feature map’s size incurs prohibitive GPU memory and computational costs. Patch-based 3D inputs reduce memory usage but compromise global spatial coherence. In contrast, purely 2D models are efficient but fail to capture inter-slice context. A pragmatic compromise is the use of 2.5D inputs [16], namely, short stacks of adjacent slices that retain some through-plane information while preserving a near-2D memory footprint.

Among the architectures that leverage 2.5D data, RNN-like networks [17] are particularly attractive. Their sequential design naturally encodes the dependencies between slices while their memory consumption remains comparable to that of standard 2D models, thereby providing volumetric context without sacrificing deployability.

In this study, we propose an ASC module that leverages prior anatomical information about the aorta to automatically enhance aortic contrast, which is designed for integration into RNN-like models. The overall network adopts an RNN-like structure to process 2.5D inputs, thereby incorporating spatial context. As each sub-model still operates on 2D inputs and only a single 2D segmenter is used, the GPU memory burden is not increased appreciably relative to the baselines. Meanwhile, the ASC module’s streamlined design enables seamless integration with common medical image segmentation backbones and significantly improves its segmentation accuracy for CT images with insufficient or no contrast.

## 2. Materials and Methods

Our proposed method is based on the assumption that, as neural networks are inspired by the structure of biological neurons, they should exhibit similar behaviors in terms of recognizing clearly enhanced structures, such as the contrast-enhanced aorta in CT images. Furthermore, incorporating certain prior knowledge can increase the prediction accuracy of neural network models. In our proposed method, such prior knowledge is represented by spatially guided automatic contrast enhancement.

### 2.1. Network Architecture

**Figure 1:**
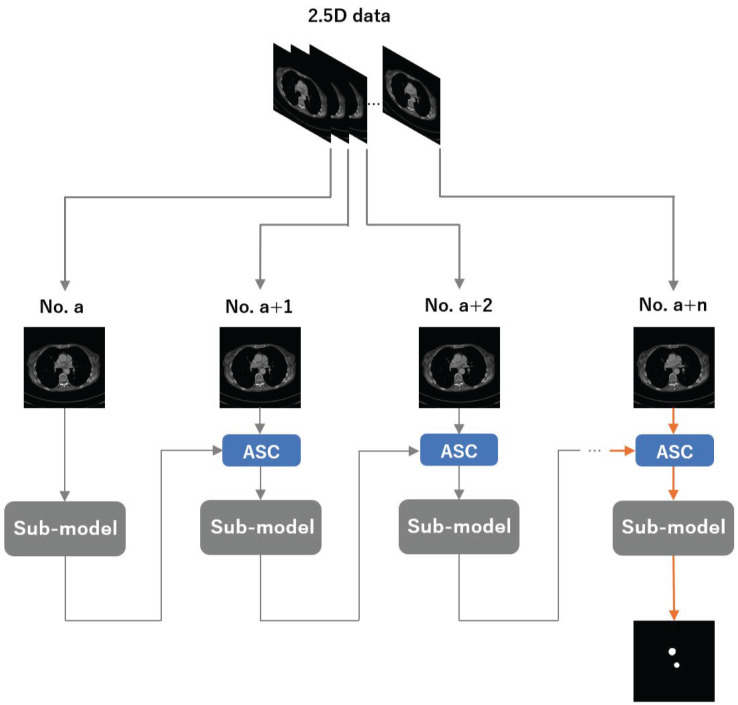
An overview of the framework presented in this study. The sub-models are the 2D segmentation models that we used as baselines. We did not modify the internal structures of these models; instead, we introduced spatial information through an RNN-like construction to enhance their segmentation performance. The orange arrows represent the flow of data for final output.

An overview of our method is shown in Figure 1. We group n adjacent CT slices into a 2.5D input, where the per-slice sub-model is a 2D segmenter (instantiated as UNet, Attention U-Net, TransUNet, or Swin-UNet). Let *a* denote the slice index. We first feed the *a*-th slice into the sub-model and obtain its segmentation. We then pass this prediction to the ASC module to construct an enhanced map, combine it with the (a+1)-th slice, and feed the enhanced input back to the same sub-model. Repeating this process (n−1) times yields the final segmentation for the target slice.

### 2.2. ASC Module

**Figure 2:**
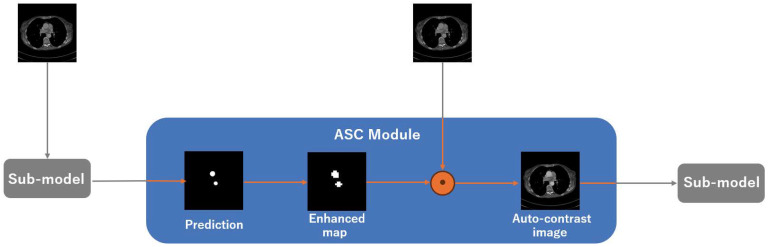
The structure of the ASC module. The sub-model in this figure is an identical 2D segmentation model.

In the ASC module (see Figure 2), the prediction for the previous CT slice is used to construct an enhanced map, which amplifies the aortic intensity in the current slice before it is fed to the sub-model, thus realizing automatic contrast enhancement. Concretely, we apply element-wise multiplication between the current CT image x and the enhanced map e_m:

(1) $$x^{\prime }=x⊙e\_m$$

where $x^{\prime }$ denotes the enhanced input that is passed to the sub-model and ⊙ indicates pixel-wise multiplication.

Some related works have applied element-wise masking (i.e., multiplying the image by a binary mask) to force the network to focus on regions of interest [17]. However, such hard masking can suppress contextual cues and inter-organ relationships. Moreover, unlike organs with well-defined boundaries such as the lungs, the aorta is difficult to delineate from surrounding tissues under insufficient contrast enhancement. Some pipelines further rely on two separate networks [18,19] (e.g., one for localization and one for segmentation) or a two-pass scheme at different resolutions, which increases computational cost and model complexity. Alternatively, soft attention methods integrate features from adjacent slices to improve target slice segmentation; however, they typically reduce interpretability and incur higher computational overhead.

**Figure 3:**
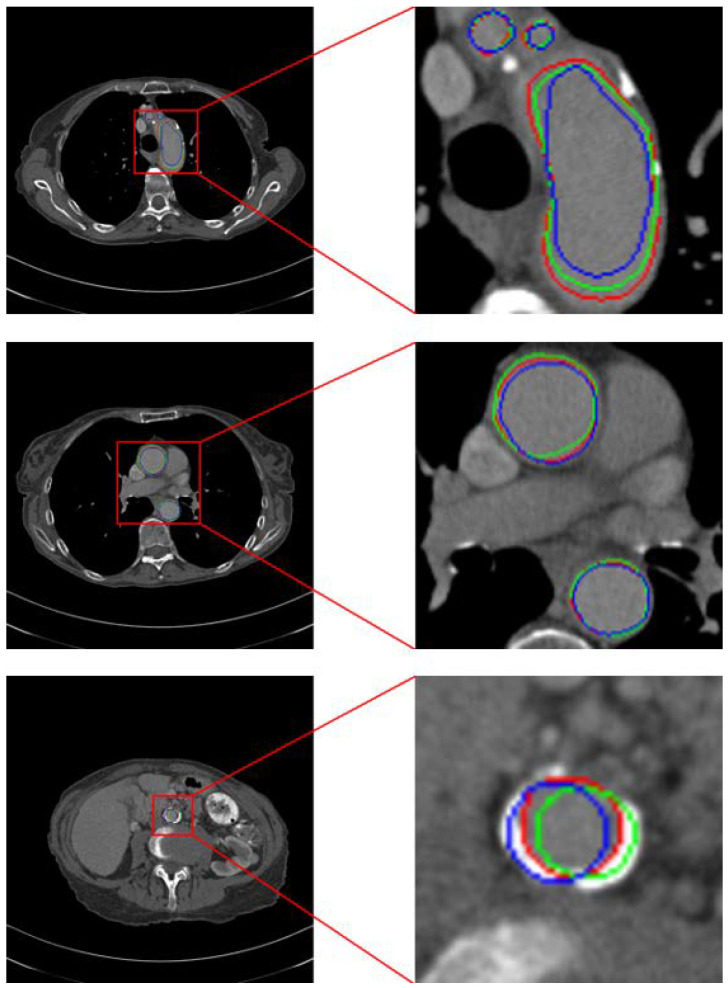
Location of aorta in CT images with similar serial numbers. The green circle is the location of the aorta in the previous CT image, the blue circle is its location in the next CT image, and the red circle indicates the location of the aorta in the central CT image.

**Figure 4:**
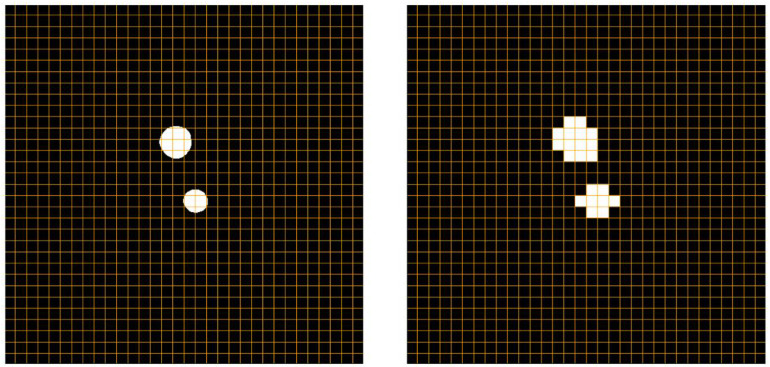
The generation of an enhanced map based on the previous prediction. We split the prediction into 16 × 16 enhancement kernels. The pixel values of areas shown in white are enhanced, while those in black areas are kept as in the original input image. This process is robust to variations in the number and morphology of aortic structures.

Therefore, we adopt a hard attention-type gating scheme to improve model performance. As shown in Figure 3, the aorta typically exhibits only minor positional shifts and generally similar morphology in adjacent CT slices. Leveraging this property, we generate an enhancement map using the previous slice’s prediction to guide the current slice. Nevertheless, small inter-slice differences remain, and the aorta’s positional offset varies across cases as a function of slice thickness. To increase the likelihood that the enhancement region covers the entire aorta despite these shifts, we expand the region using the strategy illustrated in Figure 4.

We split the previous prediction into n×n kernels and, when the average pixel value in one kernel exceeds a minimum value ϵ, the kernel value is adjusted to enhance the pixel values. Otherwise, the kernel value is adjusted to 1.0:

(2) $$KV_{e}\left(h,w\right)\left\{\begin{array}{cc} enhancement\;factor, & V_{p}\left(h,w\right)\\ 1.0, & V_{p}\left(h,w\right) \end{array}\right.$$

After performing element-wise multiplication, the pixel values in the high-confidence mask region which had been predicted as representing the aorta are enhanced.

### 2.3. Dataset

The Aortic Vessel Tree (AVT) CTA dataset [20], which includes ground-truth segmentations, was used in this study. To simulate the two cases in which CT images are optimally contrasted and poorly/non-contrasted, we split the dataset based on the CT values at the aorta locations in the CT images into two levels, as detailed in Table 1. The aorta CT values in Level 1 are over 250 [HU], while those in Level 2 are in the range of [100, 250] [HU].

### 2.4. Data Form

It is necessary to enable shuffling in the DataLoader to randomize the data and ensure that the model can readily access the input samples, thus allowing it to learn the relationships between the slices used to generate the enhanced map and the target slice to be segmented. For training, we grouped n adjacent slices into a block and fed them as input to the model.

As it is difficult to guarantee the same number of CT slices across all cases, during this process it was also ensured that valid data were always supplied within each batch. To allow the model to fully adapt to using the previous slice’s prediction to create an enhanced map for the next slice, and to enable the model to learn potential inter-slice connections, the number of slices contained in each block should not be too small. Suppose that we enclose only two slices in a block; then, only one slice is enhanced by the enhanced map, which is also the slice that is used to produce the overall prediction. In this case, the slice used to build the enhanced map remains a non-enhanced low-contrast CT image; therefore, the enhancement effect is likely to be inaccurate. As the baseline 2D models attain higher DSC and IoU values on well-contrasted CT images, the predictions on the enhanced slices within a block should become increasingly accurate. At the same time, the number of opportunities for the model to learn the relationships among the n slices in a block is (n−1); that is, to make the model more adaptable to predicting a case sequentially during inference, n should be as large as possible. On the other hand, as the time required for training and inference in our method is roughly proportional to the number of slices in each block, the number n of slices per block should not be set too large to reduce the training and inference times.

In light of the above considerations, we argue that selecting an appropriate number of slices n represents a necessary trade-off between segmentation accuracy and the computational costs of training and inference.

In our experiment, we set a block consisting of 4 CT slices to be fed into our RNN-like architecture, thus ensuring that the models have a sufficient chance to learn the relationships between slices. In particular, if one block is composed of slices from No. n to No. (n+3), the next block should be made up of slices from No. (n+1) to No. (n+4). To allow the model to process sufficient slices to automatically contrast the position of the aorta exactly, we compared the last slice’s prediction with the corresponding ground-truth mask. The resolution of each CT slice was 512×512 pixels.

### 2.5. Settings

To ensure a fair comparison, all models were trained with the same settings in the main experiments: batch size = 4, learning rate = 1 × 10^−6^, and 200 training epochs. We used the Adam optimizer. All slices within each block had a resolution of 512 × 512 pixels.

For TransUNet, the number of Transformer layers, hidden size, MLP ratio, and number of heads were set to 12, 768, 4, and 12, respectively.

For Swin-UNet, as the input resolution was 512 × 512 rather than 224 × 224, and we increased the window size from 7 to 16. As only the aorta and background were considered in this task, the number of output classes in the final segmentation head was set to 2.

For the ASC module, the enhancement kernel size was set to 16 × 16. The enhancement factor was 1.1 for Level 1 and 1.4 for Level 2.

### 2.6. Evaluation

In the experiment, we used the Dice Similarity Coefficient (DSC) as the primary evaluation metric, while the Intersection-over-Union (IoU) is reported as a complementary metric. Let $P={\left\{\;p_{i}\right\}}_{i=1}^{N}\in {\left\{0,1\right\}}^{N}$ denote the binary prediction mask and $G={\left\{\;g_{i}\right\}}_{i=1}^{N}\in {\left\{0,1\right\}}^{N}$ the corresponding ground-truth mask. Define $TP=\sum_{i=1}^{N}1\left\{p_{i}=1\wedge g_{i}=1\right\},FP=\sum_{i=1}^{N}1\left\{p_{i}=1\wedge g_{i}=0\right\}$, and $FN=\sum_{i=1}^{N}1\left\{p_{i}=0\wedge g_{i}=1\right\}$. Then, the above-mentioned metrics are computed using

(3) $$\begin{array}{cc} \mathrm{DSC}(P,G)\; & =\frac{2\;|P\cap G|}{\left|P|+|G\right|}=\frac{2\;TP}{2\;TP+FP+FN}, \end{array}$$

(4) $$\begin{array}{cc} \mathrm{IoU}(P,G)\; & =\frac{\left|P\cap G\right|}{\left|P\cup G\right|}=\frac{TP}{TP+FP+FN}. \end{array}$$

## 3. Results

### 3.1. Main Experiment

Table 2a,b compares the baselines with our method across four backbone models, reporting the results for Level 1 and Level 2 data. We observed a consistent pattern: the four baseline models performed well on Level 1 data but poorly on Level 2 data. On Level 2 data, our method achieved significant gains while keeping the parameter count essentially unchanged. Meanwhile, from Table 2a it can be observed that, in most cases, our method yielded small improvements in both DSC and IoU on Level 1 data. In contrast, TransUNet showed a slight decrease in IoU. Comparing the backbones used in this study, we believe that this discrepancy stems from the global self-attention in TransUNet’s encoder. Unlike convolutional encoders and the windowed self-attention in Swin-UNet, global self-attention together with patch tokenization may dilute fine-grained boundary cues under high-contrast conditions, leading to a minor decrease in IoU [21].

As shown in the #params column of Table 2a,b, our method introduces no additional trainable parameters. The FLOPs column further indicates that the inference cost scales approximately linearly with the number of slices processed per pass (i.e., the slices per block size). Input CT slices are 512 × 512 pixels, with numeric precision set to FP32.

In Table 2b, both the DSC and IoU scores for the baseline TransUNet and Swin-UNet models were very low. On relatively small datasets, Transformer-based models often require more training epochs than convolution-based ones to fully converge [22]. Moreover, as each block contains four slices which are processed sequentially, our proposed method effectively exposes the model to more slice-level updates per epoch. To reduce any bias due to under-training and ensure fairness, we therefore increased the training schedule of the original TransUNet and Swin-UNet models from 200 to 800 epochs; to test whether convolution-based models showed similar sensitivity, we likewise extended the original UNet’s training schedule to 800 epochs.

As shown in Table 3, the Transformer-based models achieved improved segmentation performance when the number of training epochs increased. Nevertheless, even after increasing the total training budget to approximately match that of our method, our approach still outperformed the original baselines by a substantial margin.

With our method, the change in GPU memory usage during both training and inference is negligible (see Table 4). As high-memory GPUs become increasingly expensive, achieving performance gains without increasing memory requirements is of clear practical significance.

### 3.2. Ablation Study

To evaluate the performance differences across different parameter settings and data configurations, we derived alternative training and test sets from the original datasets. To highlight the potential of our method while controlling for architectural confounders, we also assessed the segmentation accuracy of UNet—a simple, widely used baseline—under these settings.

#### 3.2.1. Effect of Number of Slices

We hypothesized that the number of slices per block is correlated with the segmentation performance. By grouping multiple slices into a single training block, we aimed to help the model to learn the relationships between the slices used to construct the enhanced map and the target slice, thereby boosting overall performance. When the block size is two, only one slice is used to generate the enhanced map, while the other serves as the target. As there is only a single enhancement step, inaccurate segmentation on the first slice may produce an enhanced map that fails to highlight the aorta, leading to sub-optimal final performance. Moreover, the model has very limited opportunities to learn inter-slice relationships.

Although the use of larger blocks (i.e., more enhancement steps) can improve performance on the target slice, our approach fundamentally trades computation time for accuracy (see Table 5). As such, including too many slices in each block leads to excessive training and inference times. Consequently, it is crucial to identify an optimal trade-off between slice count and computational cost.

Accordingly, to evaluate the impact of block size on performance, we constructed input blocks containing two, three, and four slices from the original dataset and conducted training and evaluation for each configuration.

As shown in Table 5, using blocks composed of two slices consistently yields lower segmentation accuracy in terms of both DSC and IoU. In contrast, three slices per block, even after accounting for the inherent stochasticity of deep learning, provided the best trade-off between inference speed and segmentation performance in our experiments.

Let N denote the number of pixels per CT slice and B the channel width (after the first convolution). Under a standard cost model with fixed kernel size and depth, UNet has a per-slice time complexity in the order of $O({\mathrm{NB}}^{2})$. The ASC module involves element-wise operations and small-stencil processing, contributing O(N). With B=64, the ASC cost is about $1/B^{2}=1/4096$ of the UNet cost; thus, the combined complexity per slice is

(5) $$O\left(NB^{2}\right)+O\left(N\right)\approx O\left(NB^{2}\right)$$

Empirically (see Table 5), the throughput exhibits an approximately linear dependence on the number of slices per block *m* once I/O overhead is included. Therefore, the per-block runtime scales with $O({\mathrm{mNB}}^{2})$.

#### 3.2.2. Effect of Enhancement Kernel Size and CT Density

To investigate the relationship between the CT voxel depth and enhancement kernel size, we reconstructed the Level 2 dataset using R7.nrrd as the validation set and R9.nrrd as the test set. Both volumes have an original voxel depth of 0.625 mm. We then sub-sampled along the slice (through-plane) direction by keeping every second, fourth, or eighth slice (yielding effective voxel depths of 1.25, 2.5, and 5 mm, respectively) for comparative testing. Accordingly, the enhancement kernel sizes were set to 8, 16, and 32, and each voxel-depth-specific dataset was used for both training and testing.

Table 6 summarizes the Dice and IoU metrics for the three enhancement kernels (8, 16, and 32 pixels) across the voxel density strata (high/medium/low), from which three consistent patterns emerged.

First, with ks=8, both the Dice and IoU values increased as voxel depth decreased, and the improvement was gradual within the tested range (down to 0.625 mm), without a clear performance plateau relative to the larger kernels.

Second, with ks=16, performance peaked on high-density scans (highest IoU and second-best Dice) but sharply decreased on medium and low densities; although the IoU also declined, it remained competitive at medium density, illustrating the underlying distribution of training samples across density strata.

**Figure 5:**
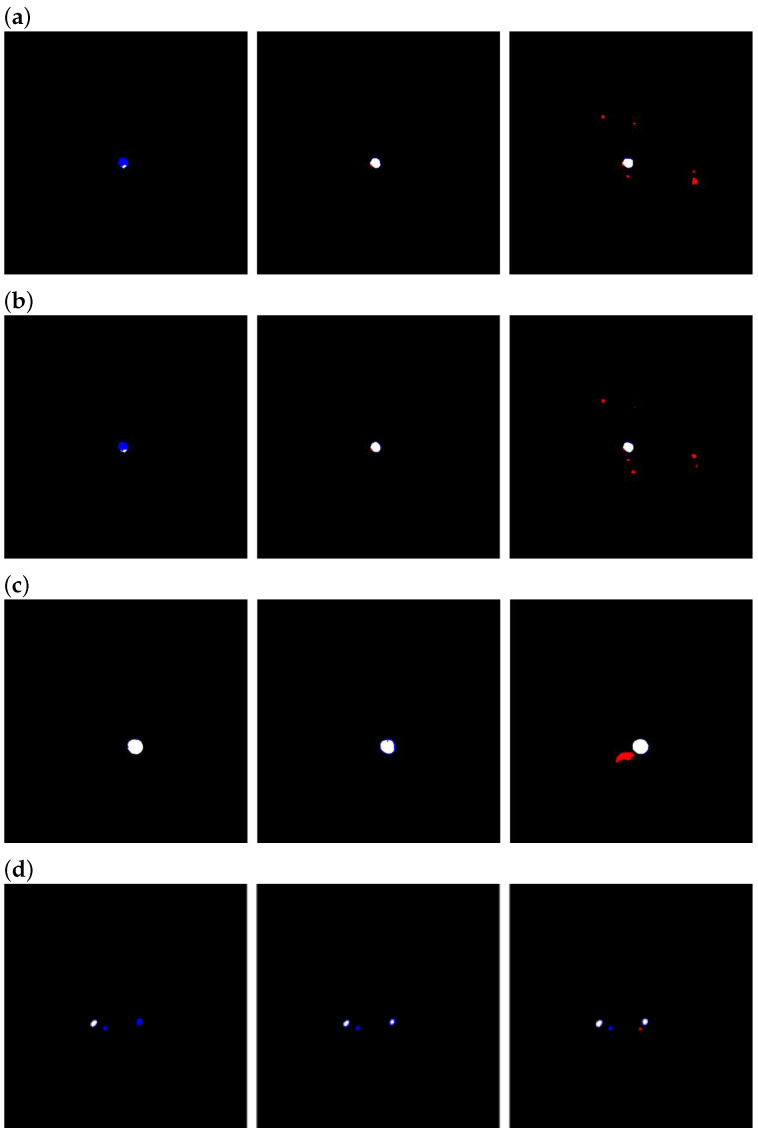
Difference maps. Black: True negatives; white: true positives; blue: false negatives; red: false positives. From left to right: enhancement kernel size = 8, 16, and 32. Rows (**a**–**d**) correspond to voxel depths of 0.625, 1.25, 2.5, and 5.0 mm, respectively.

Third, with ks=32, Dice scores remained high across all densities, whereas the IoU was lower than that for ks=16 at medium/high density, consistent with an increase in false positive areas (Figure 5).

Overall, kernel–density interaction is evident: ks=16 excels at high density but degrades on sparser slices; ks=8 improves steadily with higher density but does not clearly dominate; and ks=32 is robust in terms of Dice across densities while underperforming in IoU at medium/high density. We also observed a monotonic association between smaller voxel depth and larger gains from smaller kernels, which was consistent across cross-validation folds.

## 4. Discussion

We propose a method that effectively improves segmentation accuracy on low-contrast or non-contrast-enhanced CT scans. As shown in Table 2a, we were pleasantly surprised to observe positive effects on well-contrasted scans as well, suggesting the existence of an optimal intensity range for training deep aorta-segmentation models. In this study we used a fixed enhancement factor (EF), set to 1.1 for all Level 1 data and 1.4 for all Level 2 data, selected via validation. Using a fixed scalar avoids introducing additional networks—thereby preventing extra GPU memory overhead (see Table 4)—and preserves the overall time complexity (see Table 5). Nevertheless, even within Level 1 data, aortic intensities vary across patients and along different segments of the aorta. Because a multiplicative EF >1 amplifies such variability, the post-enhancement aortic intensity will not always fall within the presumed “optimal” range. As a future direction, we plan to adapt the ASC module and the segmentation network so that the enhancement factor becomes adaptive or learnable, enabling finer-grained enhancement of CT data while controlling the usage of GPU memory growth.

Table 7 summarizes the Dice Similarity Coefficient (DSC) and Intersection over Union (IoU) values for the UNet baseline and the UNet augmented with the ASC module, evaluated on the held-out test sets R9 and R17. Across both test sets, the ASC module yields consistent—and often substantial—improvements in segmentation performance on low/non-contrast CT scans. We acknowledge the limitation of the relatively small test sets; this study primarily introduces a methodological framework. As part of subsequent clinical validation, we will expand to multi-center datasets and conduct formal statistical significance testing to further substantiate these findings.

As a trade-off for higher accuracy, our method substantially reduced throughput relative to the baseline (see Table 2a,b). Nevertheless, even when processing a 1000-slice CT volume with the slowest configuration, that is, when using TransUNet as the sub-model, the inference time was only about 2 minutes. We consider this latency acceptable for frontline practice, particularly in clinical workflows where segmentation accuracy typically takes precedence over marginal speed.

Regarding the effect of the number of slices in the ablation study, we observed that using four slices per block yielded worse metrics than when using three slices per block. From the standpoint of limiting the training and inference times, this finding is encouraging—it suggests that the proposed ASC module and training strategy can deliver noticeable gains at a relatively small time cost, rather than requiring a larger block to chase marginal long-tailed benefits. We attribute this phenomenon to error accumulation. As the segmentations of CT slices used to generate enhanced maps are not perfectly accurate, the anticipated improvement, namely, that the enhanced map would become increasingly accurate as the number of slices per block grows, did not materialize [23].

**Figure 6:**
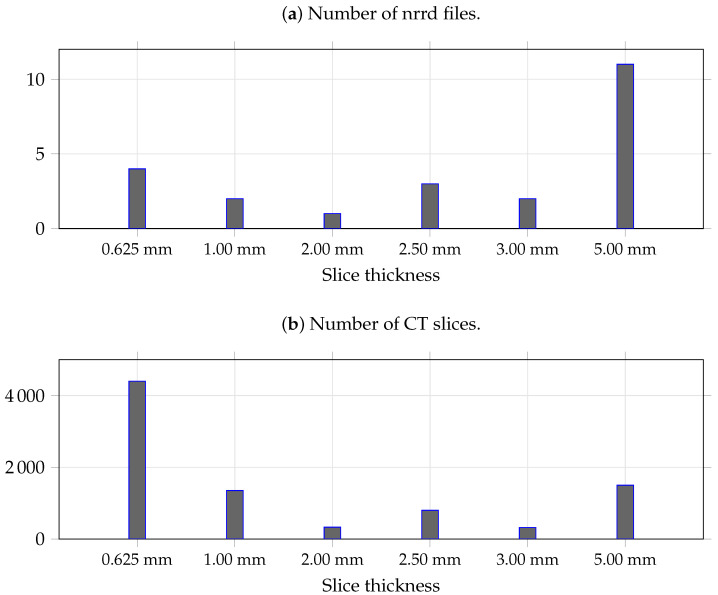
Number of CT samples at different densities. The upper panel reports the count of original NRRD volumes used for training, whereas the lower panel reports the corresponding number of extracted CT slices. The overall density distribution of the training data is noticeably imbalanced: high-density slices (small voxel depth, 0.625 and 1.00 mm) are the most prevalent and low-density slices (5.00 mm) are the next most common, while medium-density slices are comparatively scarce. The voxel depth (slice thickness) was extracted via ImageJ 1.54f Java 1.8.0_322(64-bit).

Regarding the enhancement kernel size (ks), we interpreted the observations as reflecting a balance between cross-slice alignment and background amplification. When the voxel depth was small, ks=16 yielded the highest IoU and the second-best Dice score; however, when the voxel depth was medium or large, ks=16 led to a substantial drop in both Dice and IoU values. As shown in Figure 6, our dataset has an uneven distribution of voxel depths, with noticeably fewer medium/low-density scans than high-density scans. This may indicate that ks=16 is more prone to vulnerability when there is slight misalignment between the enhancement map and the target slice.

With ks=8, the performance improved gradually as voxel depth decreased. The peak accuracy was lower than that achieved with ks=16 or ks=32; we suspect that a slice spacing of 0.625 mm is still too large for ks=8 to realize its peak performance. In addition, as a smaller enhancement kernel tends to confine mistakes within the aortic lumen rather than spuriously enhancing surrounding tissues, false positive regions remain limited and false negatives do not expand markedly as voxel depth increases. As a result, ks=8 does not suffer sharp degradation when the voxel depth grows.

In contrast, while ks=32 attained the highest Dice scores across all conditions, its IoU values on medium- and high-density data were inferior to those of ks=16. As illustrated in Figure 5, a larger kernel offers more complete coverage of the aortic region yet simultaneously amplifies nearby tissues, increasing the false positive area. As per Equation (6), the IoU penalizes false positives more stringently than Dice, which explains the divergence between these metrics. From the perspective of readability (smaller false positive area), while still seeking high overall performance, we therefore adopted ks=16 as the main experimental setting.

(6) $$\Delta {\mathrm{DSC}}_{\mathrm{FP}}=-\;\frac{2\times \mathrm{TP}}{{\left(2\times \mathrm{TP}+\mathrm{FP}+\mathrm{FN}\right)}^{2}}\;\Delta \mathrm{FP}$$

To clarify the relationship between enhancement kernel size and voxel depth more definitively, it is necessary—while acknowledging the constraints and scarcity of medical imaging data—to strive for a more balanced distribution of voxel depths in the training data without sacrificing overall sample size.

Nevertheless, as shown in Table 7, our method performs strongly not only on the high-density dataset R9 (voxel depth = 0.625 mm), where more training data are available, but also yields substantial gains on the medium-density dataset R17 (voxel depth = 2.5 mm), where the amount of available training data is smaller. We posit that although performance correlates with the enhancement kernel size and the volume of training data at a given voxel depth, the proposed ASC module can still confer benefits even when training data at similar densities are scarce. This underscores the potential of the ASC module and its associated training strategy.

## 5. Conclusions

We presented an ASC module and a training strategy grounded in CT-specific imaging characteristics, providing a more interpretable approach to improve segmentation accuracy on low-contrast scans resulting from sub-optimal contrast enhancement. From its inception, the ASC module was designed with the knowledge that the aorta is not a simple cylindrical structure, containing multiple branching and merging segments such as the ascending aorta, descending aorta, and aortic arch. Our experiments indicated that the ASC module remains robust to within-slice variability in the number and morphology of aortic cross-sections. Although this approach still warrants further investigation, for instance, by expanding the dataset to include diverse acquisition protocols and patient populations, thereby improving out-of-distribution robustness, it has already demonstrated considerable capability while adding minimal GPU memory overhead. This offers a complementary perspective to purely architecture-centric improvements and encourages reconsideration of the current trend of pursuing accuracy primarily through ever-larger models.

## Figures and Tables

**Table 1:** Dataset classification summary. D means Dongyang, K means KiTS, and R means Rider. Some nrrd files, such as D3, were unusable. The differences between the CT values of aortas in files R14, R15, R16, R18, and some other Level 2 files were unacceptably large, and their data were too limited to support the definition of an additional level; therefore, they were not used in the experiment. In our main experiment, R13 and R17 were used as the test sets for the Level 1 and Level 2 datasets, respectively.

Data Level	File Name
Level 1	D1, D2, D4, D5, D6, D7, D8, D9, D10, D11, D12, D14, D15, D16, D18, K1, K5, R5, R6, R8, R10, R11, R12, R13
Level 2	K2, K3, K4, K6, K7, K8, K9, K10, K11, K12, K13, K14, K15, K16, K17, K18, K19, K20, R1, R2, R3, R4, R7, R9, R17

**Table 2:** Results of the main experiment. Throughput denotes the output speed when using models to infer CT images. The experimental platform was equipped with an AMD Ryzen 9 9950X CPU (Advanced Micro Devices, Inc., Santa Clara, CA, USA), DDR5 5600MHz, and an NVIDIA RTX 5090 GPU (Micro-Star International Co., Ltd., New Taipei City), based on NVIDIA GPU architecture (NVIDIA Corp., Santa Clara, CA, USA). For the baseline 2D models, FLOPs were measured on a single CT slice. For the ASC-augmented setting, one forward pass involved inputting a 4-slice block and produced the last (target) slice; accordingly, the FLOPs for one complete block are taken as the FLOPs per target slice.

Model	DSC [%]	IoU [%]	#params	FLOPs	Throughput [images/s]
(**a**) Segmentation results on Level 1 data.					
UNet	82.22	79.65	34.53 M	524.53 G	32.06
UNet + ASC	**84.76**	**80.92**	34.53 M	2098.12 G	9.38
Attention U-Net	79.45	78.84	34.88 M	533.47 G	30.05
Attention U-Net + ASC	**83.90**	**80.41**	34.88 M	2133.88 G	9.11
TransUNet	84.56	**74.92**	108.44 M	384.51 G	29.36
TransUNet + ASC	**87.15**	73.13	108.44 M	1538.04 G	8.64
Swin-UNet	70.06	75.02	27.15 M	73.50 G	46.81
Swin-UNet + ASC	**80.68**	**75.91**	27.15 M	294.00 G	20.55
(**b**) Segmentation results on Level 2 data.					
UNet	61.58	55.85	34.53 M	524.53 G	32.05
UNet + ASC	**67.48**	**68.23**	34.53 M	2098.12 G	9.46
Attention U-Net	65.96	60.48	34.88 M	533.47 G	30.96
Attention U-Net + ASC	**70.82**	**67.17**	34.88 M	2133.88 G	9.14
TransUNet	49.64	30.15	108.44 M	384.51 G	27.41
TransUNet + ASC	**74.48**	**58.28**	108.44 M	1538.04 G	8.63
Swin-UNet	38.53	39.15	27.15 M	73.50 G	47.51
Swin-UNet + ASC	**61.66**	**53.66**	27.15 M	294.00 G	18.73

The bolded numbers represent better performance in the metrics.

**Table 3:** Effect of increasing the number of training epochs on segmentation accuracy on the Level 2 dataset.

Model (Epochs)	DSC [%]	IoU [%]
UNet (200)	61.58	55.85
UNet (800)	63.75	60.77
UNet + ASC (200)	**67.48**	**67.99**
TransUNet (200)	49.64	30.15
TransUNet (800)	62.89	46.46
TransUNet + ASC (200)	**74.48**	**58.28**
Swin-UNet (200)	38.53	39.15
Swin-UNet (800)	53.22	43.45
Swin-UNet + ASC (200)	**60.76**	**53.66**

The bolded numbers represent better performance in the metrics.

**Table 4:** GPU memory usage during training and inference. We performed benchmarking using one RTX 5090 GPU and the Level 2 dataset. Inclusion of the ASC module resulted in a maximum increase of 33.02 MB in GPU memory consumption during both training and inference. The increase in GPU memory consumption was at most 0.5% during training and less than 2% during inference.

Model	Train	ΔMemory	Inference	ΔMemory
UNet	8093.31 M	27.38 M ↑	2822.46 M	23.87 M ↑
UNet + ASC	8120.69 M		2846.33 M	
Attention UNet	8944.77 M	25.12 M ↑	2837.38 M	26.10 M ↑
Attention UNet + ASC	8969.89 M		2863.48 M	
TransUNet	9517.31 M	33.02 M ↑	2578.69 M	23.63 M ↑
TransUNet + ASC	9550.33 M		2602.32 M	
Swin-UNet	6086.64 M	30.75 M ↑	1376.16 M	23.69 M ↑
Swin-UNet + ASC	6117.39 M		1399.85 M	

Up arrows represent an increase in GPU memory utilization.

**Table 5:** Comparison of UNet model’s segmentation results, throughput, and overall time complexity (Big-O) for different numbers of slices per block. N=H×W denotes the number of pixels in the input CT slice and B denotes the number of channels in the feature map after the first convolutional layer.

Slices per Block	DSC [%]	IoU [%]	Throughput [images/s]	Overall Time Complexity
2	61.78	65.64	17.94	$O({\mathrm{NB}}^{2})$
3	69.17	69.11	12.56	$O({\mathrm{NB}}^{2})$
4	67.48	68.23	9.46	$O({\mathrm{NB}}^{2})$

**Table 6:** Segmentation results using different enhancement kernel sizes on CT data of varying voxel depth.

Voxel Depth [mm]	Kernel Size	DSC [%]	IoU [%]
0.625	8	71.81	68.73
	16	77.41	**77.54**
	32	**82.51**	68.64
1.25	8	71.54	68.43
	16	77.34	**77.92**
	32	**82.89**	68.80
2.5	8	70.70	68.06
	16	65.75	**71.39**
	32	**83.04**	69.33
5.0	8	70.13	68.56
	16	64.98	70.88
	32	**83.42**	**71.22**

The bolded numbers represent better performance in the metrics.

**Table 7:** Comparison of DSC/IoU: UNet vs. UNet+ASC on R9 and R17 test sets.

Test Case	DSC (Base)	DSC (ASC)	ΔDSC	IoU (Base)	IoU (ASC)	ΔIoU	Voxel Depth [mm]
R9	60.77	77.41	16.64	55.03	77.54	22.51	0.625
R17	61.58	67.48	5.9	55.85	68.23	12.38	2.5

## Data Availability

The example code implementing the ASC Module proposed in this work has been made publicly available on GitHub at: [https://github.com/academic-owl/ASC-Module–Preliminary-Study.git].

## Abbreviations

The following abbreviations are used in this manuscript:

CT	Computed Tomography
ASC	Automatic Spatial Contrast
IoU	Intersection-over-Union
DSC	Dice Similarity Coefficient
GPU	Graphics Processing Unit
CNN	Convolutional Neural Network
